# A modified particle swarm optimization algorithm for parameter estimation of a biological system

**DOI:** 10.1186/s12976-018-0089-6

**Published:** 2018-11-05

**Authors:** Raziyeh Mosayebi, Fariba Bahrami

**Affiliations:** 1 School of Electrical and computer Engineering, College of Engineering, University of Tehran, Tehran, Iran; 2 CIPCE, School of Electrical and Computer Engineering, College of Engineering, University of Tehran, Tehran, Iran

**Keywords:** Biological System Modeling, Iterative UKF, Particle Swarm Optimization, Simulated Annealing

## Abstract

**Background:**

Mathematical modeling has achieved a broad interest in the field of biology. These models represent the associations among the metabolism of the biological phenomenon with some mathematical equations such that the observed time course profile of the biological data fits the model. However, the estimation of the unknown parameters of the model is a challenging task. Many algorithms have been developed for parameter estimation, but none of them is entirely capable of finding the best solution. The purpose of this paper is to develop a method for precise estimation of parameters of a biological model.

**Methods:**

In this paper, a novel particle swarm optimization algorithm based on a decomposition technique is developed. Then, its root mean square error is compared with simple particle swarm optimization, Iterative Unscented Kalman Filter and Simulated Annealing algorithms for two different simulation scenarios and a real data set related to the metabolism of CAD system.

**Results:**

Our proposed algorithm results in 54.39% and 26.72% average reduction in root mean square error when applied to the simulation and experimental data, respectively.

**Conclusion:**

The results show that the metaheuristic approaches such as the proposed method are very wise choices for finding the solution of nonlinear problems with many unknown parameters.

## Background

The parameter estimation of a biological model is a crucial step of a system description. These models are an approximation of the real phenomenon and some of their parameters do not have physical interpretation and their presence is to compensate the reductions and approximations in the model. These approximations often stem from the lack of our knowledge about the biological system.

The modelling of biochemical pathway is possible with the concurrent measurement of biochemicals. This is the result of latest developments in data acquisition technologies which provide us with abundance of time profiles of metabolites or proteins that can be used for mathematical modeling of biochemical networks [1].

The first step to mathematically model these phenomena is to specify a comprehensive framework that represent the underlying structure and convey the thorough information. The second step is to choose a promising approach to find a global solution for the unknown parameters of the model.

Frequently, the problem of biological parameter estimation are said to be NP hard, so they are multi-modal and ill conditioned i.e. there are more than one true solutions for the estimated parameters that fit the model and produce the time course information [2]. Many algorithms have been developed to address this problem. Among these algorithms, the gradient optimization methods often fail to reach the true solution due to its high sensitivity to initialization [3]. The heuristic algorithms however have been shown to be able to solve the NP hard problems and overcome the problem of multimodality and achieve the global optimality [4].

Many researchers have investigated the problem of parameter estimation in biological systems. As the results of their works, algorithms with promising outcomes have been emerged. EKF, IUKF, alternating regression, simulated annealing, firefly, and genetic algorithms are some example [5–10]. In [11], the author has developed an improved particle filter method for estimation of the parameters of an S-system, and shows the superiority of this method over the ordinary particle filter algorithm from the accuracy and convergence rate point of view. Besides this method, Ding and et al. [12] have used a kalman filter based least square method for state estimation of canonical state space problem and a decomposition technique for enhancing the computational time. In [13], Moles and et al. have compared the global optimization methods for the problem of biochemical pathway modeling. In their paper, the authors have shown that the evolutionary algorithms such as meta-heuristic methods are the most proper choices for solving the NP hard problems and preventing from local solutions. Some other papers have employed the evolutionary algorithms. For example, in [14], the author developed a hybrid firefly algorithm to estimate the parameter of a highly complex and nonlinear biological model. This method uses a neighborhood search by utilizing the evolutionary methods and compares its results in aspects of accuracy and computational time with the firefly and nelder-mead algorithms. In [15], the authors have demonstrated the capability of the evolutionary algorithms for estimation of the unknown parameters of a system in a reasonable amount of time. They proposed a parallel implementation of an enhanced Differential Evolution (DE) using Spark to reduce the computational time of the search process by including a selected local search and exploiting the available distributed resources. In [16], the author has modified the UKF algorithm to estimate the parameters of an E-coli system. The method is called Iterative UKF and is designed to prevent from the early saturation of the filter gain. As a result the estimation error is decreased in comparison with the ordinary UKF algorithm.

One of the main problems of the parameter estimation of complex and highly nonlinear models is the capability of the method to estimate the global solution. In these models, as the fitness function is non-convex, the number of the local solutions is large. This is the main reason of the algorithms such as the gradient base methods and optimal filtering that are unable to find the global solution, since these algorithms are highly sensitive to the initialization of the unknown parameters [3]. On the other hand, the evolutionary methods such as particle swarm optimization are powerful methods for exploring the search space in order not to stick in local solutions.

In this paper, a modified PSO algorithm is presented. In this method, a decomposition technique is employed so that the algorithm has higher ability for the exploitation technique near the final solution. The improvement of the exploitation technique results in minor movements of the particles near the global solution and prevents from larger jumps and deterioration of the value of fitness function. As a result, the method has less mean square error compare with the ordinary PSO algorithm.

The modified PSO algorithm is proposed for estimation of the parameters of a simulated biological model from a synthetic data [16]. This pathway generated data is modelled by a canonical model which is made by the S-system. Then, this algorithm is employed for real data of the E-coli system [15, 16]. The results of the algorithm are then compared with the IUKF, SA and PSO algorithms in Root Mean Square Error point of view. We have demonstrated that the novel PSO algorithm is accomplished to estimate the true parameters and has less RMSE compare with the SA and IUKF and the ordinary PSO algorithm.

This paper is organized as follows. The mathematical modelling is defined in section II. The parameter estimation methods and the proposed technique are described in section III. The simulated and experimental results are presented in section IV and the paper is finally concluded in Section V.

## Mathematical modelling

### Review stage

Consider a nonlinear dynamical model for a biological system as below:

1 $$ \dot{x}(t)=f\left(x(t),u(t),w\right) $$

where *x* ∈ *ℝ*^*N*^ is the metabolites, *u* ∈ *ℝ*^*m*^ is the concentration vectors of biomolecules that is the vector of independent variables, and *w* ∈ *ℝ*^*q*^ is the parameter vector. The problem is to find a solution for the unknown parameters that are best fitted to the model.

Different canonical modelling frameworks are available to describe a biological phenomenon. In this paper, among some frameworks such as Lotka-Volterra [17], S-systems [18], and cooperative and saturation models [19], the S-system is used to express the nonlinear model of eq. (1)

2 $$ {\dot{x}}_i={\alpha}_i\prod \limits_{j=1}^{N+m}\prod \limits_{k=1,k\ne j}^{N+m}{x}_j^{g_{ij}}{x}_k^{g_{ik}}-{\beta}_i\prod \limits_{j=1}^{N+m}\prod \limits_{k=1,k\ne j}^{N+m}{x}_j^{h_{ij}}{x}_k^{h_{ik}},\kern1.25em i=1,2,\dots, N, $$

where *α*_*i*_ > 0 and *β*_*i*_ > 0 are rate coefficients and *g* and *h* are kinetic orders. The number of dependent variables is *N* which represents the metabolism and *m* is the number of independent variables that are considered as inputs to the system.

Considering the eq. 2, we have *x* = [*x*_1_, …, *x*_*N*_]^*T*^as the state vector, *u* = [*x*_*N* + 1_, …, *x*_*N* + *m*_]^*T*^ as the input vector and *w* = [*α*_1_, …, *α*_*N*_*β*_1_, …, *β*_*N*_, *g*_11_, …, *g*_*N*, *N* + *m*_, *h*_11_, …, *h*_*N*, *N* + *m*_]^*T*^as the parameter vector, while *q* = *N*(2 + 2(*N* + *m*)) is the largest number of the unknown parameters. Many algorithms have been developed to estimate the unknown parameters of such nonlinear equations. In section III, some of these methods are presented.

## Methods

In order to compare the performance of the proposed method with another heuristic approach, the Simulated Anealling method is used. Moreover, for the further analysis of heuristic methods, a non heuristic approach based on Unscented Kalman filter is applied. These methods besides the proposed algorithm are described in this section.

### Iterative Kalman filter (IUKF)

The Unscented Kalman Filter (UKF) is a modified framework of the Kalman Filter for nonlinear modelling. In this filter, one or both the process and measurement equations might be nonlinear and the nonlinear kalman filter is required. The UKF algorithm employs the unscented transform as a nonlinear transformation to compute the statistics of a random variable.

The UKF algorithm is briefly explained as follow:

Consider the discrete state space model of eq. I:

3 $$ x\left[k+1\right]=F\left(x\left[k\right],u\left[k\right],w\right), $$

By the definition of*y*[*k*] = *x*[*k* + 1], *k* = 0, …, *N* − 1, the above equation can be rewritten as the following nonlinear process equation:

4 $$ y\left[k\right]=F\left(x\left[k\right],u\left[k\right],w\right), $$

where *x* and *u* are the inputs, *y* is the output and *w* is the unknown parameter with dimension *q* that is to be estimated. To estimate the parameters, the following state space representation is defined as follows:

5 $$ {\displaystyle \begin{array}{l}w\left[k+1\right]=w\left[k\right]+r\left[k\right]\\ {}y\left[k\right]=F\left(x\left[k\right],u\left[k\right],w\left[k\right]\right)+e\left[k\right],\end{array}} $$

where the first model is the process equation, driven by *r* as the process noise. The latter is the measurement equation driven by the input and the measurement noise *e*. the UKF algorithm is able to estimate the parameter *w* based on the following pseudo code:

1. Initialize the unknown parameter and the covariance matrix

6 $$ {\displaystyle \begin{array}{l}\kern0.5em \widehat{\omega}\left[0\right]=E\left[\omega \right]\\ {}\kern0.5em {P}_{\omega \left[0\right]}=E\left[\left(\omega -\widehat{\omega}\left[0\right]\right){\left(\omega -\widehat{\omega}\left[0\right]\right)}^T\right]\end{array}} $$

2. Assuming the parameter vector *ω* with mean $\overline{\omega}$ and covariance *P*_*ω*_ a set of 2q + 1 sigma vectors $\mathcal{W}$ are obtained through the following equations

7 $$ {\displaystyle \begin{array}{l}{P}_{\omega \left[k\right]}^{-}={P}_{\omega \left[k-1\right]}+{R}^r\left[k-1\right]\\ {}W\left[k|k-1\right]=\left[{\widehat{\omega}}^{-}\left[k\right],{\widehat{\omega}}^{-}\left[k\right]+\gamma \sqrt{P_{\omega \left[k\right]}^{-}},{\widehat{\omega}}^{-}\left[k\right]-\gamma \sqrt{P_{\omega \left[k\right]}^{-}}\right],\end{array}} $$

where $\gamma =\sqrt{q+\lambda }$, *λ* = *ε*^2^(*q* + *ϗ*) − *q* is a scaling parameter, and $\Big({\sqrt{\left(q+\lambda \right){P}_{\omega}\Big)}}_i$ represents the ith column of the matrix. The constant 10^−4^ < *ε* < 1 controls the sigma points spread around $\overline{\omega}$. *ϗ* is also a scaling parameter, which is regularly fixed to 0 or 3 − *q* and *q* is the dimension of parameters [14].

3. The sigma points are transformed with the nonlinear process *F*

8 $$ \mathcal{D}\left[k|k-1\right]=F\left(x\left[k\right],u\left[k\right],\mathcal{W}\left[k|k-1\right]\right) $$

4. The mean and covariance of the transformed sigma point of step 3 are calculated as follows

9 $$ \widehat{d}\left[k\right]={\sum \limits}_{i=0}^{2q}{W}_i^{(m)}{\mathcal{D}}_i\left[k|k-1\right] $$

10 $$ {P}_{\widehat{d},\widehat{d}}\left[k\right]={\sum}_{i=0}^{2q}{W}_i^{(c)}\left[{D}_i\left[k\left|k-1\right.\right]-\widehat{d}\left[k\right]\right]{\left[{D}_i\left[k\left|k-1\right.\right]-\widehat{d}\left[k\right]\right]}^T+{R}^e\left[k\right], $$

Where $$ {W}_i^{(m)}={W}_i^{(c)}=\frac{1}{2\left(\lambda +q\right)} $$,$$ {W}_0^{(m)}={W}_0^{(c)}=\frac{\lambda }{\left(\lambda +q\right)} $$.

5. The cross covariance matrix of the measurement and parameter vectors are calculated

11 $$ {P}_{\omega, \widehat{d}}\left[k\right]={\sum}_{i=0}^{2q}{W}_i^{(c)}\left[{\mathcal{W}}_i\left[k|k-1\right]-{\widehat{\omega}}^{-}\left[k\right]\right]{\left[{D}_i\left[k|k-1\right]-\widehat{d}\left[k\right]\right]}^T $$

6. The kalman gain, parameters and the covariance matric is then updated

12 $$ \mathcal{K}\left[k\right]={P}_{\omega, \widehat{d}}\left[k\right]{P}_{\widehat{d},\widehat{d}}^{-1}\left[k\right] $$

13 $$ \widehat{\omega}\left[k\right]= Proj\left[\widehat{\omega}\left[k\right]+\mathcal{K}\left[k\right]\left(d\left[k\right]-\widehat{d}\left[k\right]\right)\right] $$

14 $$ \kern4em {P}_{\omega}\left[k\right]={P}_{\omega \left[k\right]}^{-}-\mathcal{K}\left[k\right]{P}_{\widehat{d},\widehat{d}}\left[k\right]{\mathcal{K}}^T\left[k\right] $$

In [16], the authors have shown that the convergence rate of the UKF algorithm is small and the filter might not converge to the true parameters. The main reason is the early saturation of the kalman gain resulting from the assignment of not suitable initial values. Thus, the small filter gain results in small changes in the estimation of parameters and the algorithm converge very slowly.

To remedy this problem, the authors have developed the IUKF algorithm. In this algorithm, after applying the UKF algorithm, RMSE is calculated through the following equation

15 $$ RMSE=\sqrt{\sum_{k=0}^{n-1}{\left(y\left[k\right]-\widehat{y}\left[k\right]\right)}^2} $$

where *y*[*k*] is the true measurement and $\widehat{y}\left[k\right]$ is the estimated output. If *MSE* is smaller than a threshold value *δ*_*E*_ or the iteration number of the algorithm is large enough the algorithm will stop, otherwise the UKF algorithm will be initialized with the final estimate of the parameters and their covariance which produces better initial values for the UKF algorithm. If the difference of two consecutive RMSE is less than a threshold value *δ*_*R*_, the covariance matrix resets to the first initial value preventing from small changes in covariance matrix.

### Simulated annealing

SA algorithm is a meta-heuristic algorithm introduced by Scott Kirkpatrick and et al. [20]. This algorithm is based on heating and cooling of the materials, starting with a prior solution and improves it in a repetitive process to reach the optimized solution for the problem. It consists of an inner and outer loop. The inner loop displaces the last solution in a solution space with a local search and updates the obtained solution based on a probabilistic criterion. The outer loop decreases the temperature of the process consistently. This temperature affects the performance of the inner loop. In the beginning with high temperature, the algorithm performs well in searching the solution space and prevents from the local solution in non-convex problems. By decreasing the temperature, it demonstrates a good capability in exploration.

At the first iterations that the temperature is high, in the contrary to the non-proper value of the cost function, the probability of a bad solution in the inner loop is high. This property prevents the algorithm from convergence in local responses. In the last iterations of the outer loop with the loose of temperature, the probability of a bad solution is decreased and the most proper solution is chosen with high probability.

### Particle swarm optimization

Particle Swarm Optimization, also called PSO, is a population based stochastic optimization method intruduced by Kennedy and Eberhart [21]. PSO simulates the activities of school of birds, swarms of insects or groups of fish, in which individuals are called particles and the population is named a swarm. In this method, a position *P* and a velocity *V* are assigned to each particle. These particles are scattered around in the search-space based on a few procedures. The particles are scattered following their own best known position P^*^, in the search-space as well as the total swarm’s best known position ${P}_g^{\ast }$. The velocity of the *j*th dimension of particle *i* in iteration *t*, is obtained as follow:

16 $$ {\displaystyle \begin{array}{l}{V}_{ij}(t)=W.{V}_{ij}\left(t-1\right)+{c}_1{r}_1\left({P}_{ij}^{\ast}\left(t-1\right)-{P}_{ij}\left(t-1\right)\right)\\ {}\kern1.92em +{c}_2{r}_2\left({P}_{gj}^{\ast}\left(t-1\right)-{P}_{ij}\left(t-1\right)\right)\end{array}} $$

Where *W* is the inertia weight and is normally in the range of 0.9–1.2 [22], *c*_1_ and *c*_2_ are constants that are set to 2, and *r*_1_ and *r*_2_ are randomly generated stochastic parameters in the interval [0, 1].

The new position of the *i*th particle is then updated by the following formula:

17 $$ {P}_{ij}(t)={P}_{ij}\left(t-1\right)+{V}_{ij}(t) $$

The PSO algorithm is known as a fast simple method, suitable for non-convex NP-hard problems such as biological pathway modelling. Despite its great performance in exploring the solution space, it has weakness in exploiting the optimal solution when reaching to its neighborhood. To cope with this deficiency, the researchers usually adopt some heuristics to the algorithm or hybridize it with some other methods. In this paper, a novel heuristic is embedded in the PSO algorithm to improve its exploiting capabilities. Here, we apply a decomposition technique in a sequential platform with the canonical PSO. The proposed algorithm, named as DPSO, has a two-phase structure. The first phase is a standard PSO, where the algorithm finds a solution in the neighborhood of the optimal one, known as the Global-Best. In order to enhance the capability of the first phase in reaching near the optimal solution as close as possible, the inertia weight in the eq. (16) is linearly reduced in each iteration of the PSO algorithm, ending with a final value equal to 0.1. Using this technique provides the algorithm with more precision in the final iterations, when the particles are in the neighbor of the best solution. The second phase, utilizes a decomposition technique as following pseudo-code:

For *i* = 1: Iteration Number

For j = 1:Param_Number

- Relocate the position of all the particles as the global-best
- Scatter the particles in the *j*th dimension in the neighbor of the global-best
- Find the optimal value of the *j*th dimension using the PSO algorithm
- Update the global-best

endfor.

endfor.

As described in the above pseudo-code, the second phase of the algorithm has an inner loop where it tries to find the optimal value of all the decision variables, considering the values of the others as constant values, using the canonical PSO. In the second phase, we needed to be more precise in our searching method. So, we concentrated the ability of the PSO algorithm from a multi-dimensional search space to a single-dimension exploitation: by decomposing the problem into each decision variable, the PSO was able to search the optimal value of each decision variable near the vicinity of the best found solution at hand, while considering other decision variables as fixed values. This searching technique is repeated for all decision variables. In the outer loop, the whole procedure is repeated to insure evading from a local solution. Using this technique allows the method to exploit the optimal solution with higher levels of accuracy, resulting in a reliable high quality solution.

## Results

### Simulations

In this section, we demonstrate the capability of the modified PSO algorithm with two synthetic simulations. In the first simulation, no additive noise has been assumed. In the second simulation, additive white Gaussian noise with SNR = 20 is considered. The data are generated through the following model:

18 $$ {\displaystyle \begin{array}{l}{\dot{x}}_1={\alpha}_1{x}_3^{g_{12}}{x}_5^{g_{15}}-{\beta}_1{x}_1^{h_{11}}\\ {}{\dot{x}}_2={\alpha}_2{x}_1^{g_{21}}-{\beta}_2{x}_2^{h_{22}}\\ {}{\dot{x}}_3={\alpha}_3{x}_2^{g_{32}}-{\beta}_3{x}_3^{h_{33}}{x}_4^{h_{34}}\\ {}{\dot{x}}_4={\alpha}_4{x}_1^{g_{41}}-{\beta}_4{x}_4^{h_{44}}\end{array}} $$

and the parameters are as follows:

19 $$ {\displaystyle \begin{array}{l}\omega =\Big[{\alpha}_1,{\alpha}_2,{\alpha}_3,{\alpha}_4,{\beta}_1,{\beta}_2,{\beta}_3,{\beta}_4,{g}_{13},{g}_{15},{g}_{21},{g}_{32},{g}_{41},\\ {}\kern1.2em {h}_{11},{h}_{22},{h}_{33},{h}_{34},{h}_{44}\Big],\end{array}} $$

Table 1 shows the chosen parameters of the above model.

**Table 1:** The chosen Parameters of the simulated S-system

True parameters	*α*_*i*_	*g*_*i*1_	*g*_*i*2_	*g*_*i*3_	*g*_*i*4_	*β*_*i*_	*h*_*i*1_	*h*_*i*2_	*h*_*i*3_	*h*_*i*4_
*x*_1_	20.0	0	0	−0.8	0	10.0	0.5	0	0	0
*x*_2_	8.0	0.5	0	0	0	3.0	0	0.75	0	0
*x*_3_	3.0	0	0.75	0	0	3.0	0	0	0.5	0.2
*x*_4_	2.0	0.5	0	0	0	6.0	0	0	0	0.8

**Fig. 1:**
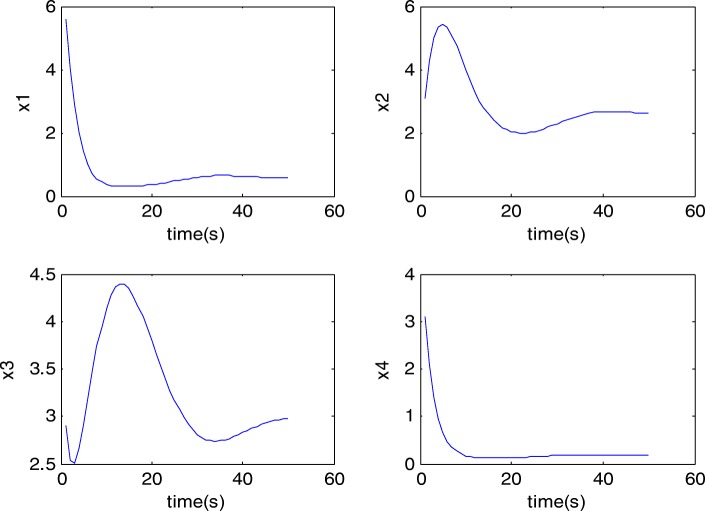
time profile of the four synthetic states: Four random initial values are generated to produce the four synthetic metabolisms according to eq. 18. These data is used for estimation of the parameters of the assumed model. RMSE is then computed between the data obtained by the estimated model and the true data

Four random initial values are generated to produce the four synthetic metabolisms. The time profiles of these four synthetic states are obtained from eq. (18) and shown in Fig. 1.

**Fig. 2:**
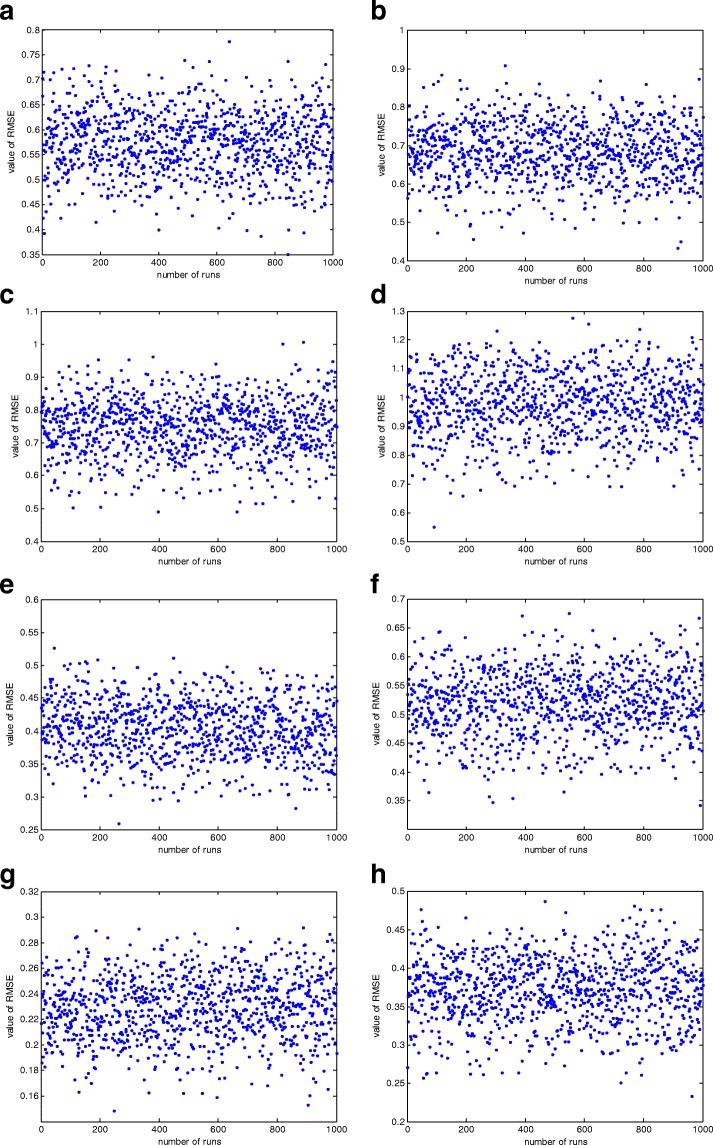
the RMSEs of 1000 simulation runs for noise free and noisy scenarios. The figures in left column represent the noise free scenario in **a** IUKF, **c** SA, **e** PSO, **g** DPSO. The figures in the right column correspond to noisy scenario in **b** IUKF, **d** SA, **f** PSO and **h** DPSO

Tables 2 through 5 depict the results obtained from IUKF, SA, PSO and the proposed DPSO algorithms. In these tables, the average of estimated parameters and the corresponding average RMSE for each algorithm in 1000 simulations are presented. As it is obvious from the tables, DPSO algorithm has higher capabilty for estimating the unknown parameters in both simulation scenarios. The RMSE of the proposed method is less than the other three algorithms. These simulations demonstrate the enhancement of the exploitation phase in the PSO algorithm employing the proposed technique compared with the ordinary PSO and also the capability of the method for estimation of more precise parameters compared with the IUKF and SA algorithms. Figure 2 illustrates the value of RMSEs of noise free and noisy scenarios in each 1000 simulation runs for each algorithm in Tables 2, 3, 4 and 5.

**Table 2:** The estimated parameters of two simulations for IUKF algorithm

True parameters	*α*_*i*_	*g*_*i*1_	*g*_*i*2_	*g*_*i*3_	*g*_*i*4_	*β*_*i*_	*h*_*i*1_	*h*_*i*2_	*h*_*i*3_	*h*_*i*4_	RMSE
IUKF noise free measurement											0.5698
*x*_1_	20.4897	0	0	−0.3092	0	10.488	0.9954	0	0	0	
*x*_2_	7.5180	1.5027	0	0	0	3.4884	0	1.7552	0	0	
*x*_3_	3.4989	0	1.7556	0	0	2.4842	0	0	1.5019	0.7053	
*x*_4_	2.4902	0.9887	0	0	0	6.4926	0	0	0	1.294	
IUKF noisy measurement with SNR 20											0.6853
*x*_1_	19.6459	0	0	−0.1437	0	9.643	1.6490	0	0	0	
*x*_2_	9.1521	1.1415	0	0	0	3.6681	0	1.4249	0	0	
*x*_3_	3.6506	0	1.8956	0	0	3.6517	0	0	1.1638	0.8546	
*x*_4_	2.6518	1.6585	0	0	0	7.6445	0	0	0	1.4447

**Table 3:** The estimated parameters of two simulations for SA algorithm

True parameters	*α*_*i*_	*g*_*i*1_	*g*_*i*2_	*g*_*i*3_	*g*_*i*4_	*β*_*i*_	*h*_*i*1_	*h*_*i*2_	*h*_*i*3_	*h*_*i*4_	RMSE
SA noise free measurement											0.7471
*x*_1_	21.6611	0	0	−0.1552	0	10.65	1.1561	0	0	0	
*x*_2_	8.6545	1.1575	0	0	0	3.629	0	1.4052	0	0	
*x*_3_	3.6379	0	1.4034	0	0	3.6475	0	0	1.1439	0.8504	
*x*_4_	1.6503	1.1432	0	0	0	6.6653	0	0	0	1.4568	
SA noisy measurement with SNR 20											0.9762
*x*_1_	18.8551	0	0	0.05911	0	9.8271	1.3714	0	0	0	
*x*_2_	8.8778	1.3444	0	0	0	4.8634	0	1.5988	0	0	
*x*_3_	3.8245	0	1.6078	0	0	5.8395	0	0	1.3836	1.0416	
*x*_4_	2.8509	1.3273	0	0	0	6.8551	0	0	0	1.6388

**Table 4:** The estimated parameters of two simulations for the PSO algorithm

True parameters	*α*_*i*_	*g*_*i*1_	*g*_*i*2_	*g*_*i*3_	*g*_*i*4_	*β*_*i*_	*h*_*i*1_	*h*_*i*2_	*h*_*i*3_	*h*_*i*4_	RMSE
PSO noise free measurement											0.4040
*x*_1_	20.3569	0	0	−0.4444	0	10.3510	0.8602	0	0	0	
*x*_2_	8.3541	0.858	0	0	0	3.3464	0	1.1024	0	0	
*x*_3_	3.3513	0	1.1049	0	0	3.3478	0	0	0.8554	0.5437	
*x*_4_	2.3560	0.8419	0	0	0	6.3490	0	0	0	1.148	
PSO noisy measurement with SNR 20											0.5160
*x*_1_	16.0762	0	0	−0.3411	0	7.4571	0.9537	0	0	0	
*x*_2_	8.4461	1.9448	0	0	0	3.4396	0	1.1908	0	0	
*x*_3_	1.453	0	1.1971	0	0	3.4558	0	0	0.9496	0.6451	
*x*_4_	2.4434	0.9482	0	0	0	8.4386	0	0	0	1.2565

**Table 5:** The estimated parameters of two simulations for the DPSO algorithm

True parameters	*α*_*i*_	*g*_*i*1_	*g*_*i*2_	*g*_*i*3_	*g*_*i*4_	*β*_*i*_	*h*_*i*1_	*h*_*i*2_	*h*_*i*3_	*h*_*i*4_	RMSE
DPSO noise free measurement											0.2291
*x*_1_	20.2023	0	0	−0.5991	0	10.2041	0.7005	0	0	0	
*x*_2_	8.1990	0.6944	0	0	0	3.2005	0	0.9511	0	0	
*x*_3_	3.2062	0	0.9527	0	0	3.2002	0	0	0.6936	0.3995	
*x*_4_	2.1953	0.7009	0	0	0	6.1974	0	0	0	0.9952	
DPSO noisy measurement with SNR 20											0.3722
*x*_1_	21.326	0	0	−0.4736	0	10.3255	0.8217	0	0	0	
*x*_2_	8.3162	0.8279	0	0	0	3.3227	0	1.0783	0	0	
*x*_3_	3.3122	0	1.0662	0	0	3.3331	0	0	0.8197	0.5247	
*x*_4_	2.3317	0.8094	0	0	0	5.3346	0	0	0	1.1281	

The mean percentage improvement of DPSO algorithms over SA, IUKF and PSO methods is 60.06% and 48.72% with respect to noise free and noisy scenarios.

### Real data

In order to compare the performance of the algorithms in an experimental scenario, a real dataset is utilized from the Cad system. The Cad system is one of the conditional stress response modules in *E. coli*, which is induced only at low pH and a lysine-rich environment [23, 24]. The following S-system can be used to model this phenomenon

20 $$ {\displaystyle \begin{array}{l}\frac{d\left[ CadA\right]}{dt}={\alpha}_1{\left[ CadBA\right]}^{g_{12}}-{\beta}_1{\left[ CadA\right]}^{h_{11}}\\ {}\frac{d\left[ CadBA\right]}{dt}={\alpha}_2{\left[ Lys\right]}^{g_{24}}{\left[{H}^{+}\right]}^{g_{25}}-{\beta}_2{\left[ CadBA\right]}^{h_{22}}\\ {}\frac{d\left[ Cadav\right]}{dt}={\alpha}_3{\left[ CadA\right]}^{g_{31}}{\left[ Cadav\right]}^{g_{33}}{\left[ Lys\right]}^{g_{34}}\\ {}\frac{d\left[ Lys\right]}{dt}=-{\beta}_4{\left[ CadA\right]}^{h_{31}}{\left[ Cadav\right]}^{h_{33}}{\left[ Lys\right]}^{h_{34}}\end{array}} $$

The description and the parameters of the model have obtained from [16]. The parameters of this system have been estimated by the above mentioned methods. Then, these parameters will be used to generate the time profiles of the states of the Cad system in eq. (20). To compare the results of the algorithms, the RMSE is calculated as the error of the generated time profiles and the real datasets obtained by the measurements. Table 6 shows the resultant RMSEs.

**Table 6:** The RMSE of the three algorithms in real data experiment

Algorithms	IUKF	SA	PSO	DPSO
RMSE	1.049	1.119	0.893	0.741

**Fig. 3:**
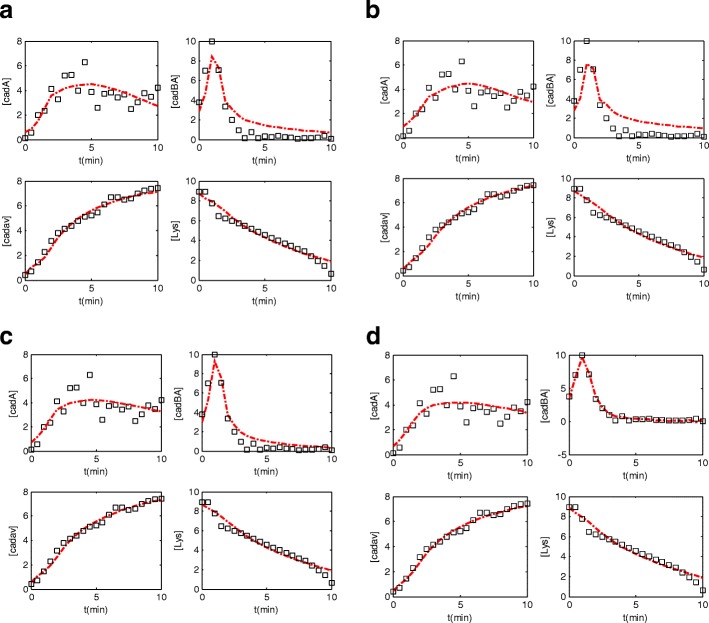
: the estimated time profile of the true data set with the three algorithms: **a** IUKF, **b** SA, **c** PSO, **d** DPSO: the red color shows the estimated time course and the squares represent the true data points. DPSO algorithm has less RMSE compared with the other algorithms and it can be inferred from the figure. The estimated time profile of DPSO method follows the variations of the true values more precisely

The generated time profiles obtained by the estimated parameters along with the true time profiles are depicted in Fig. 3.

The proposed decomposition technique is applied on the ordinary PSO to have smaller movements near the global solution and prevent from larger jumps that deteriorate the results. Therefore, as it is explained earlier, the DPSO algorithm has higher capability in exploitation step compared with the ordinary PSO algorithm. In the real dataset experiment, the RMSE of DPSO algorithm is 0.741 revealing the capability of the proposed method in comparison to IUKF and SA and PSO algorithms. For this real data experiment, the percentage improvement of DPSO algorithm over SA, IUKF and PSO methods is 29.36%, 33.78%, 17.02%, respectively.

## Discussion

Four different algorithms have been discussed in this paper. IUKF has an analytical approach and it provides the solution under the mathematics of kalman filter. However, the heuristic approaches are very popular in the literature for the nonlinear problems with large number of parameters. Therefore, two metaheuristic approaches are considered besides IUKF. SA and PSO algorithms are two powerfull methods for finding the best solution in NP hard problems. The proposed DPSO algorithm is developed to have higher capability in exploitation step. As a result this algorithm has less RMSE compared with the other three algorithm due to smaller jumps near the global solution. The results show the superiority of DPSO algorithms over the other three methods in RMSE point of view.

In simulations, a model with 17 parameters is considered and two scenarios with different SNR have been analysed. RMSE is computed with the estimated and the true parameters. The RMSE of DPSO is less than the other three algorithms as it is expected.

In real experiment scenario the data of CAD system is used to estimate the paramters of an assumed model for the time profile of the involved metabolisms. The model is similar to the one in simulation scenarios and the parameters are estimated. The DPSO method has less RMSE between the true data and the data obtained from the model with the estimated parametrs. This accuracy is obtained as a result of more running time compared with the PSO and SA algorithms. However, this time is not a significant problem because this methods are offline and a model is estimated to be used for further analysis. The running time of each algorithm is 343, 305, 287 and 416 s for IUKF, SA, PSO and DPSO algorithms, respectively.

## Conclusion

In this paper, a PSO based algorithm is suggested to estimate the unknown parameters of the nonlinear biological models. The proposed method is compared against IUKF, SA and the ordinary PSO algorithm. The results show the superiority of the DPSO algorithm compared with the other three methods. The results of the simulation and experimental scenarios reveal the capability of the proposed algorithm for estimation of the unknown parameters of a nonlinear biological system.

## References

[1] Harrigan GG, Goodacre R, eds. Metabolic profiling: its role in biomarker discovery and gene function analysis. Springer Science & Business Media, 2012.

[2] Gu X (2016). A multi-state optimization framework for parameter estimation in biological systems. IEEE/ACM Trans Comput Biol Bioinform.

[3] Friedl G, Kuczmann M (2014). Population and gradient based optimization techniques, a theoretical overview. Acta Technica Jaurinensis.

[4] Doerner KF, Maniezzo V (2018). Metaheuristic search techniques for multi-objective and stochastic problems: a history of the inventions of Walter J. Gutjahr in the past 22 years. CEJOR.

[5] Meskin N, et al. Parameter estimation of biological phenomena modeled by s-systems: An extended kalman filter approach. Decision and Control and European Control Conference (CDC-ECC), 2011 50th IEEE Conference on. IEEE, 2011.

[6] Chou I-C, Martens H, Voit EO (2006). Parameter estimation in biochemical systems models with alternating regression. Theor Biol Med Model.

[7] Daniels BC, Nemenman I (2015). Efficient inference of parsimonious phenomenological models of cellular dynamics using S-systems and alternating regression. PLoS One.

[8] Wang C, Zhang L. Comparison of parameters estimation methods based on the systems biology model of breast cancer. Progress in Informatics and Computing (PIC), 2015 IEEE International Conference on. IEEE, 2015.

[9] Gonzalez OR (2006). Parameter estimation using simulated annealing for S-system models of biochemical networks. Bioinformatics.

[10] Chou I-C, Voit EO (2009). Recent developments in parameter estimation and structure identification of biochemical and genomic systems. Math Biosci.

[11] Mansouri MM (2014). State and parameter estimation for nonlinear biological phenomena modeled by S-systems. Digital Signal Process.

[12] Ding F, Liu X, Ma X (2016). Kalman state filtering based least squares iterative parameter estimation for observer canonical state space systems using decomposition. J Comput Appl Math.

[13] Moles CG, Mendes P, Banga JR (2003). Parameter estimation in biochemical pathways: a comparison of global optimization methods. Genome Res.

[14] Abdullah A (2013). An evolutionary firefly algorithm for the estimation of nonlinear biological model parameters. PLoS One.

[15] Teijeiro D (2017). A cloud-based enhanced differential evolution algorithm for parameter estimation problems in computational systems biology. Clust Comput.

[16] Meskin N (2013). Parameter estimation of biological phenomena: an unscented Kalman filter approach. IEEE/ACM Trans Comput Biol Bioinform.

[17] Skvortsov A, Ristic B, Kamenev A (2018). Predicting population extinction from early observations of the Lotka–Volterra system. Appl Math Comput.

[18] Meskin N, Nounou H, Nounou M, Datta A, Dougherty ER. Parameter estimation of biological phenomena modeled by S-systems: an extended Kalman filter approach: Proc IEEE 50th Decision and Control and European Control Conf (CDC-ECC); 2011. p. 4424–9.

[19] Sorribas B, Hernndez-Bermejo EV, Alves R (2007). Cooperativity and saturation in biochemical networks: a Saturable formalism using Taylor series approximations. Biotechnol Bioeng.

[20] Kirkpatrick S., Gelatt C. D., Vecchi M. P. (1983). Optimization by Simulated Annealing. Science.

[21] Eberhart R, Kennedy J. A new optimizer using particle swarm theory. In: Micro machine and human science, 1995. MHS’95, Proceedings of the sixth international symposium on: IEEE; 1995.

[22] Shi Y, Eberhart RC. A modified particle swarm optimizer: Proc. of IEEE World Conf. on Computation Intelligence; 1998. p. 69–73.

[23] Küper C, Jung K. CadC-mediated activation of the cadBA promoter in Escherichia coli. J Mol Microbiol Biotechnol. 2005;10(1):26–9. 10.1159/000090346 16491024

[24] Fritz Georg, Koller Christiane, Burdack Korinna, Tetsch Larissa, Haneburger Ina, Jung Kirsten, Gerland Ulrich (2009). Induction Kinetics of a Conditional pH Stress Response System in Escherichia coli. Journal of Molecular Biology.

